# Using 3D Printing Technology to Design Split‐Piece Sleeve Prosthesis in the Revision Surgery of Tumor‐Type Total Elbow Prosthetic Fractures: A Case Report

**DOI:** 10.1111/os.14070

**Published:** 2024-04-17

**Authors:** Kai Zhai, Kai Zheng, Ming Xu, Zhe‐ming Bao, Zi‐wei Hou, Xiu‐chun Yu

**Affiliations:** ^1^ Department of Orthopedics The 960th Hospital of the PLA Joint Logistice Support Force Jinan China

**Keywords:** 3D Printing, Elbow joint tumor, Limited revision, Prosthesis fracture, Split‐piece sleeve prosthesis

## Abstract

**Background:**

Revision of tumor‐type prosthetic fractures is very challenging in clinical work. Traditional repair methods may not be able to meet the needs of complex cases or cause greater bone damage. Therefore, more effective and reliable solutions need to be found.

**Case Presentation:**

This study presents a novel revision technique for managing fractures of tumor‐type total elbow prostheses. A 57‐year‐old female patient was diagnosed with a left distal humeral bone tumor accompanied by pathological fracture and underwent customized tumor‐type total elbow prosthesis arthroplasty. After 5 years, she experienced pain and encountered difficulty in flexing the left elbow while lifting heavy objects. The X‐ray examination revealed a fracture of the distal humeral prosthesis. As a response, the elbow joint was initially explored, and the damaged component of the prosthesis was extracted. Subsequently, we utilized 3D printing technology to design a split‐piece sleeve prosthesis and effectively restored the fractured left distal humerus implant. During the 2‐year follow‐up, The X‐ray demonstrated satisfactory positioning of the prosthesis, which remained securely affixed without any indications of loosening. The Mayo Elbow Performance Score (MEPS) reached 80 points, the Musculoskeletal Tumor Society (MSTS) attained a score of 28 points, and the range of motion of the elbow was measured between 25° and 110°, revealing favorable functional outcomes.

**Conclusion:**

The utilization of a 3D printed split‐piece sleeve prosthesis presents a viable clinical treatment strategy for addressing fractures in tumor‐type elbow prostheses.

## Graphical abstract

A 57‐year‐old female patient was diagnosed with a left distal humeral bone tumor accompanied by pathological fracture and underwent customized tumor‐type total elbow prosthesis arthroplasty. After 5 years, the X‐ray examination revealed a fracture of the distal humeral prosthesis (A). The damaged components of the prosthesis are extracted (B). Using 3D printing technology to design split‐piece sleeve prosthesis (C). The radiographs obtained during the 2‐year follow‐up demonstrated optimal prosthesis positioning without any evident signs of implant loosening, periprosthetic fracture, or breakage (D).

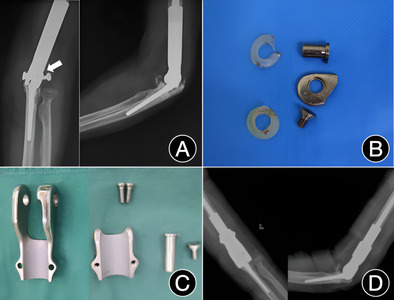

## Background

Elbow bone tumors account for approximately 1% of the overall incidence of bone tumors.^1^ Due to the complex anatomy of the elbow, it is an important location for nerves and blood vessels. Performing a simple tumor segment resection, inactivation, or replantation, as well as tumor curettage and internal fixation, may potentially result in complications, such as elbow instability, nerve damage, harm to surrounding muscles and soft tissues, and an increased risk of tumor recurrence. Amputation or elbow disarticulation not only affects the patient's quality of life, but also places a psychological burden on the patient. Advancements in medical treatments like radiotherapy and chemotherapy, along with the evolution of elbow prosthetics, have made limb‐sparing surgery a viable option. Elbow prostheses have evolved from simple single‐axis hinges to more advanced unrestricted or semi‐restricted designs through extensive research.^2^ Customized restrictive elbow prostheses are frequently utilized in the treatment of elbow tumors, and the incidence of prosthetic breakage among postoperative complications is significantly lower than aseptic loosening, nerve damage, and infection.^3^ Revision surgery for prosthetic fracture is crucial to restore elbow function and the patient's quality of life. Traditional revision surgery may result in significant bone damage and pose challenges in achieving satisfactory outcomes for the elbow. Therefore, it is necessary to explore a more effective and reliable approach. This study presents a case demonstrating the utilization of 3D printing technology for the development of a split‐piece sleeve prosthesis for repairing fractures in tumor‐type elbow joint prostheses. The principles, clinical performance, and clinical effects of this technology were discussed, and its advantages and limitations were evaluated. Through the exploration and summarization of split‐piece sleeve technology's application in the revision surgery of tumor‐type total elbow prosthetic fractures, the aim of this study was to furnish clinicians with more valuable information about this technology, improving patient treatment.

### Case Presentation

**Figure 1:**
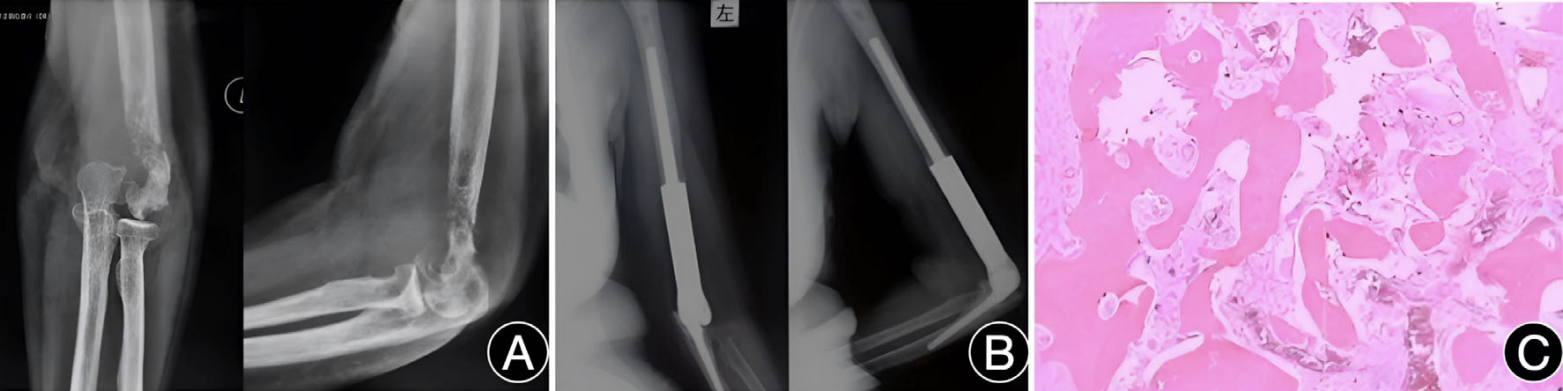
A 56‐year‐old female patient underwent tumor‐type total elbow arthroplasty for a pathological fracture of the left distal humerus.

A 57‐year‐old female patient was admitted to the hospital on October 14, 2016, due to pain in her left upper arm and limited movement for half a month due to trauma. An X‐ray image of the left elbow after admission showed bone destruction in the distal left humerus (Figure 1A). No obvious malignant tumor cells were found in puncture pathology. As the bone of the distal humerus was severely damaged when the patient was first admitted to the hospital, and it was difficult to preserve the elbow, tumor‐type prosthetic replacement of the left elbow was performed. The customized elbow joint prosthesis is manufactured by Chunli Zhengda Company. The metallic components are fabricated using TC4 forged titanium alloy, while the non‐metallic parts consist of medical‐grade ultra‐high molecular weight polyethylene (The model is CLDZ‐02). Postoperative review of the anteroposterior and lateral views of the left elbow showed that the prosthesis was in an appropriate position (Figure 1B). Pathology suggested left distal humeral hemangioma with active chondroblast proliferation in the focus area. Immunohistochemical staining results indicated negativity for P53, CD, and Ki‐67 (Figure 1C). The incision was healed in the first stage, the elbow function was recovered well, and regular follow‐up was given. Five years after the surgery, the patient suddenly suffered from left elbow pain and difficulty in flexing the elbow when lifting heavy objects. Anteroposterior and lateral X‐ray images of the left elbow showed that the prosthesis was broken. The patient was diagnosed with a prosthetic fracture following tumor‐type total elbow prosthesis arthroplasty upon readmission to the hospital. She had a body temperature of 36.5°C, height of 1.57 m, weight of 56 kg, and a body mass index (BMI) of 22.7 kg/m^2^. Physical examination revealed significantly limited range of elbow joint motion and mild non‐radiating pain upon percussion at the elbow joint. Sensation and muscle tone in both upper limbs were found to be normal.

### Treatment Process

**Figure 2:**
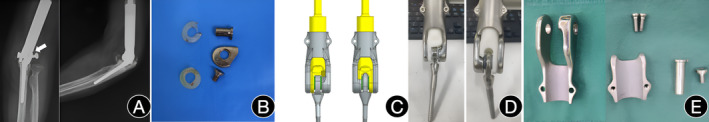
3D printing design and purpose of split‐piece sleeve prosthesis.

Preoperative planning: Following the patient's admission to the hospital, X‐ray and computed tomography (CT) scan images revealed a fracture in the humeral prosthesis located externally at the hinge (Figure 2A).The intramedullary bone cement stems in the humerus and ulna demonstrated no evidence of loosening, and there was an absence of fracture or osteolysis surrounding the prosthesis, thereby confirming its inherent stability. Following precise identification of the fracture location, we opted to develop a novel revision prosthesis, referred to as the split‐piece sleeve prosthesis, utilizing 3D printing technology. The elbow joint was initially explored prior to the revision surgery, with the aim of conducting a comprehensive investigation. Subsequently, the damaged components of the prosthesis are meticulously extracted on the lateral side of the elbow joint to assess the extent of the inflicted harm (Figure 2B). Given the absence of any looseness in the humeral intramedullary stem, a comprehensive revision of the prosthesis would inevitably result in more severe trauma. In line with the principle of limited revision, a customized prosthesis was developed for the purpose of revision. This prosthesis is primarily utilized to encase the distal broken part and restore the stability of the hinge. Based on the patient's original data, Chunli Zhengda Company utilized 3D printing technology to create a resin model of the original prosthesis post‐fracture and a split‐piece sleeve prosthesis made of TC4 forged titanium alloy (Figure 2C,D). After a period of 10 days, we successfully acquired the prosthesis, which was further divided into distinct anterior and posterior components. Notably, the fracture end of the original prosthesis was reinforced with a thickened design to effectively occupy the defective area and ensure comprehensive coverage around the humerus. The distal prosthesis was then affixed to the contact surface of the original prosthesis using bone cement, and the screws were securely fastened. Subsequently, the distal end of the sleeve prosthesis was connected to the forearm bone (ulna and radius) prosthesis in order to facilitate stable motion (Figure 2E).

**Figure 3:**
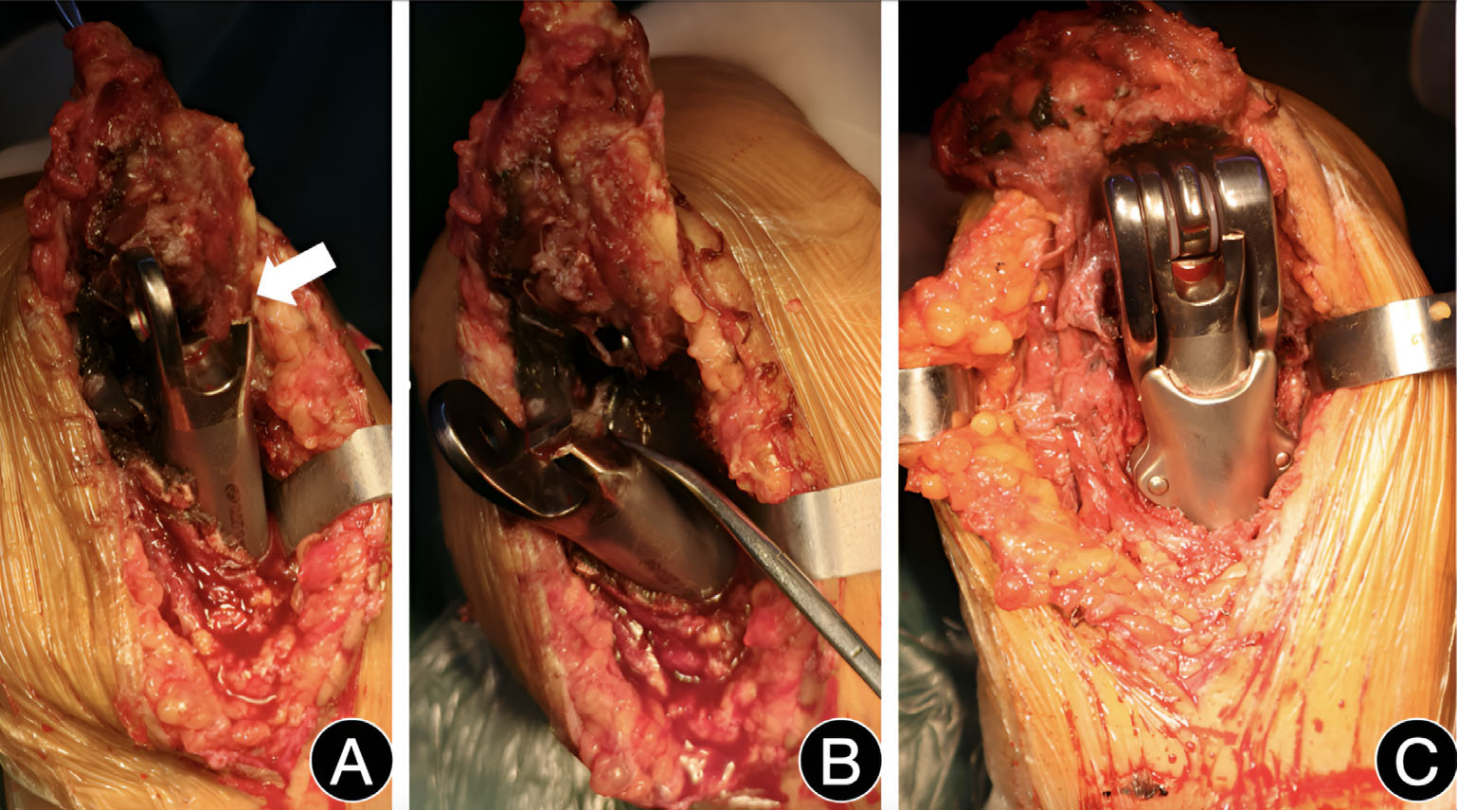
Implementation of split‐piece sleeve prosthesis in revision surgery.

Surgical procedure: The patient was positioned supine, and a tourniquet was applied proximally at the root of the left upper arm. The surgical area was routinely disinfected and draped. A longitudinal incision was made on the posterior median plain of the left elbow, about 20 cm long, and the triceps brachii was exposed layer by layer. The tongue‐shaped muscle flap was turned distally to expose the ulnar nerve on the ulnar side of the distal humerus. The ulnar nerve was completely exposed and released and then pulled to the front for protection. The soft tissue around the prosthesis of the distal humerus and proximal ulna was released and pushed forward. The hinge device of the prosthesis was peeled off to reveal black boundary membrane tissue around the prosthesis, and the distal humeral prosthesis was partially broken (Figure 3A,B). The boundary membrane tissue was cleaned, the fixation bolt was removed, and a custom‐made sleeved humeral condylar prosthesis was installed on a trial basis. The fixation bolt and polyethylene pad were utilized to reduce the elbow. When the size of the prosthesis was found to be appropriate and tight, the elbow was dislocated, the bone cement was adjusted, the screws were tightened, and it was then attempted to reset the elbow again (Figure 3C). The elbow joint prosthesis is securely fitted, enabling a normal range of motion in the elbow joint. The surgical incisions were meticulously closed in a layered manner, followed by the placement of negative pressure drainage tubes and application of sterile dressings.

Postoperative treatment: The administration of antibiotics both preoperatively and postoperatively was implemented as a prophylactic measure against potential infection. The analgesic approach incorporates the principle of multimodal combined analgesia. Drainage tube was extracted 48 h postoperatively. An external fixation brace was employed to immobilize the elbow in a functional position within a period of 3 weeks post‐surgery.

### Outcome and Follow‐Up

**Figure 4:**
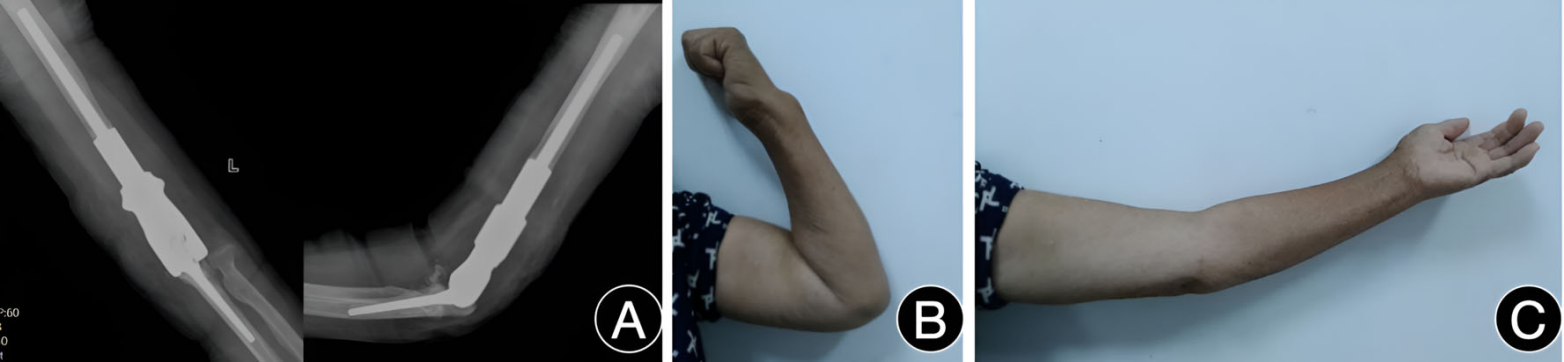
Prosthesis condition and left elbow joint function 2 years post‐surgery.

The patient underwent outpatient follow‐up visits at 1, 3, and 6 months post‐surgery for a duration of 2 years, and no postoperative complications were observed during the this period. Outpatient follow‐up comprised a comprehensive physical examination and assessment of anteroposterior and lateral X‐ray images of the left elbow. The X‐ray at the last follow‐up demonstrated satisfactory positioning of the prosthesis without any evident signs of loosening, periprosthetic fracture, or breakage. (Figure 4A).The patient's elbow function was effectively improved, and the elbow range of motion was approximately 25°–110° (Figure 4B,C). The Mayo Elbow Performance Score (MEPS) achieved a score of 80 points. The Musculoskeletal Tumor Society (MSTS) attained a score of 28 points. The patient is currently asymptomatic, with a Visual Analog Scale (VAS) score of 0, and has successfully resumed activities of daily living.

## Discussion

Bone or soft tissue tumors of the elbow have been relatively rarely reported. Before the 1970s, amputation was recognized as the mainstream surgical method for treating malignant tumors of the elbow.^4^ However, with the advancement of medical technology, the reconstructive surgery following the resection of malignant elbow tumors has undergone years of progress and evolution. Initially, the methods mainly relied on fusion or resection without reconstruction, while these methods limited the patient's elbow function and reduced the patient's quality of life. Subsequently, as bioengineering technology was continually explored, the design, materials, and implantation techniques of elbow prostheses have seen continuous improvement. The developed prostheses can better simulate the physiological structure and function of the elbow, thus, modern elbow reconstruction after malignant tumor resection can now not only accomplish tumor removal, but also partially restore the elbow's function and stability.^5^ At present, an emerging interdisciplinary field arising from the convergence of modern computer technology and orthopedic medicine, known as digital orthopedics, utilizes tools such as three‐dimensional model reconstruction, 3D printing, personalized osteotomy guides, finite element analysis, computer‐aided navigation, virtual reality, augmented reality, robot‐assisted surgery, and artificial intelligence. These applications aim to offer patients more precise and personalized diagnosis and treatment plans.^6^, ^7^ In a study conducted by Iwamoto *et al*.^8^ 28 patients with elbow osteoarthritis or rheumatoid arthritis underwent preoperative non‐hinge total elbow replacement following digital orthopedic planning. During the surgery, the elbow prosthesis was accurately placed and the surgical cost was reduced, leading to decrease of the incidence of postoperative complications and improvement of mid‐ and long‐term efficacy. In a study conducted by Liang *et al*.^9^ 3D printing technology was applied to patients with bone tumors in the distal humerus or proximal ulna, and the mentioned study involved half‐elbow reconstruction following tumor segment resection. A total of 13 patients had no serious postoperative complications and obtained satisfactory elbow mobility and clinical efficacy.

Although tumor‐based elbow prostheses have achieved encouraging clinical results in the treatment of elbow tumors, their complication rates are higher than those of other replacement surgeries.^10^ The overall complication rate in 47 patients reported in Casadei *et al*.^4^ study was 30%. In 33 patients assessed by Kruckeberg *et al*.^5^ the overall complication rate was as high as 46%. The most common complications included nerve damage, aseptic loosening, periprosthetic fractures, infection, etc.^10^ The fracture of elbow tumor‐type prosthesis is relatively rare, however, there have been reports of failures in the hinge mechanism (Table 1). Mohammed *et al*.^11^ reported a case involving a patient with a fractured elbow prosthesis hinge and humeral fracture. In lieu of opting for total humeral replacement, they performed hemi‐shoulder arthroplasty and prosthetic fracture revision using intramedullary humeral needles. This approach effectively preserved humeral bone mass and minimized injury to the surrounding muscle attachment points. Pham *et al*.^12^ reported a case of failure in the shaft assembly of the Coonrad‐Morrey elbow prosthesis. The axle and the polyethylene bushings were replaced during the initial revision surgery, but a year later, the axle failed again. The elbow prosthesis was effectively repaired through the utilization of a custom‐designed locking axle. William *et al*.^13^ reported that among the 83 patients, five demonstrated central locking and bushing component failure, with two of these cases experiencing subsequent secondary failure and undergoing revision using a more robust central axis equipped with a lock washer and set screw. Common causes of prosthetic fractures include fatigue fracture of metal materials, uneven stress distribution of the prosthesis, and excessive tumor resection. The case reported in this article caused the prosthesis to break due to external force. To date, there is a paucity of literature documenting the techniques employed in revision surgery for fractures of elbow prostheses. Traditional revision surgery requires removal of the prosthesis, which may cause greater trauma and serious complications to the patient.^14^ In this study, 3D printing technology was employed for the management of revision surgery following hinge failure resulting from fracture in tumor‐type elbow prostheses. Considering the absence of cement stem loosening in the humerus and ulnar bone marrow, as well as the lack of fracture and osteolysis around the prosthesis. The split‐piece sleeve prosthesis was designed based on the principle of limited revision. Firstly, the damaged components of the prosthesis are carefully extracted to evaluate the extent of the inflicted damage. Subsequently, leveraging the patient's primary data and advanced 3D printing technology, a meticulously split‐piece sleeve prosthesis is developed. The prosthesis exhibited a seamless compatibility with the original ruptured prosthesis during the surgical procedure. The prosthesis was used in the revision surgery of this patient. The main purpose is to reduce surgical difficulties, including difficulty in removing the prosthesis and easy peri‐prosthetic fractures during surgery, in order to restore the function and stability of the elbow, while attenuating surgical trauma to the patient. After 2 years of effective follow‐up, the patient did not encounter any complications, such as nerve damage or loosening of the split‐piece sleeve prosthesis, nor did they experience hinge failure. Furthermore, the stability and partial range of motion of the elbow joint were successfully restored.

**TABLE 1:** Clinical report of limited revision surgery in the elbow joint.

Author	Year	Number of cases	Fracture location	Modalities of revision surgery	Follow‐up	Outcome
Mohammed	2016	1	Fracture of peri‐prosthetic and hinge mechanism	Intramedullary humeral replacement	6 months	None complication, satisfactory function
Pham	2014	1	Hinge mechanism	Locking axle	3 years	None complication, satisfactory function
Seitz	2009	2	Hinge mechanism	Heavy‐duty central bolt with locked washer	_	_

Using 3D printing technology for the design of split‐piece sleeve prosthesis in revision surgery of tumor‐type total elbow prosthetic fractures offers numerous advantages in terms of surgical precision and patient outcomes. Firstly, the split‐piece sleeve prosthesis advantage lies in its exceptional adaptability to prosthetic fractures occurring at various anatomical sites. Particularly in cases where the intramedullary needle remains stable without any signs of loosening, yet a fracture occurs at the distal end of the prosthesis. Secondly, the split‐piece sleeve technology can effectively address the limitations associated with conventional revision surgeries, offering enhanced adaptability and adjustability. Mitigate the necessity for extensive revision surgeries, while obviating the intraoperative removal of intramedullary bone cement, thereby diminishing the incidence of periprosthetic fractures. Thirdly, in the event of a reoccurrence of fracture in the split‐piece sleeve prosthesis, it can be readily and efficiently replaced once again. Nevertheless, split‐piece sleeve technology exhibits certain limitations. Firstly, the complexity of the surgical procedure necessitates a high level of expertise and experience on the part of the osteo‐oncologist. Secondly, the patient engagement in effective communication is crucial prior to surgical intervention, encompassing the necessity of exploratory surgery preceding revision surgery, the duration of waiting for prosthesis preparation and potential postoperative complications. Thirdly, this prosthesis is secured using screws and bone cement, while the hinge remains affixed with a polyethylene pad. Consequently, we expect that the wear rate of the prosthesis will be in accordance with that of the original implant, however, additional long‐term follow‐up observations are imperative to elucidate the implant's sustained durability. In addition, this method of repair is subject to certain limitations based on factors such as the patient's age and ability to undergo surgical intervention, as well as an evaluation of the patient's skeletal condition prior to surgery. In cases of severe bone loss or significant implant subsidence, it may be necessary to consider alternative revision strategies. The use of split‐piece sleeve revision prostheses may also be limited depending on factors such as tumor type and location. In summary, the split‐piece sleeve technology, as an innovative repair method, has broad application prospects in the revision surgery of tumor‐type total elbow prosthetic fractures. Through individualized repair plans, limited revision concepts and reduction of surgical trauma, clinicians can assist patients to recover their elbow function and provide better surgical results.

### Prospect of Clinical Application

With the advancement and implementation of 3D printing technology, the design and production of personalized prostheses has become more precise and efficient. This technological progress has provided technical support for the development and production of customized split‐piece sleeve prosthesis, enabling the prostheses to better accommodate the specific needs of each patient. Additionally, the split‐piece sleeve prosthesis is capable of retaining the firmly fixed intramedullary stem of the original prosthesis for limited revision. This feature reduces the complexity of surgery and promotes rapid recovery of limb function, ultimately enhancing surgical success rates and patient satisfaction.

## Conclusions

In conclusion, the successful design of the split‐piece sleeve prosthesis benefits from the preservation of the patient's original prosthesis data and utilization of 3D printing technology. The retrieval of the fractured components of the prosthesis prior to revision surgery is strongly recommended in order to achieve a precise and personalized prosthesis design that ensures reliable restoration of the original implant. This technology offers a pragmatic solution for revision surgery of tumor‐type prosthetic fractures.

## Conflict of Interest Statement

All authors declare that they have no conflicts of interest concerning this study.

## Author Contribution

Kai Zhai wrote the manuscript; Kai Zheng collected the data and reviewed the manuscript; Ming Xu has collated the images and completed follow‐up visits; Zhe‐ming Bao and Zi‐wei Hou collected associated references; Xiu‐chun Yu conceived the study and made the final revision to the manuscript. All authors read and approved the manuscript.
